# A 3-Month, Randomized, Double-Blind, Placebo-Controlled Study Evaluating the Ability of an Extra-Strength Marine Protein Supplement to Promote Hair Growth and Decrease Shedding in Women with Self-Perceived Thinning Hair

**DOI:** 10.1155/2015/841570

**Published:** 2015-03-25

**Authors:** Glynis Ablon

**Affiliations:** Ablon Skin Institute Research Center, Manhattan Beach, CA 90266, USA

## Abstract

An oral marine protein supplement (MPS) is designed to promote hair growth in women with temporary thinning hair (Viviscal Extra Strength; Lifes2good, Inc., Chicago, IL). This double-blind, placebo-controlled study assessed the ability of MPS to promote terminal hair growth in adult women with self-perceived thinning hair associated with poor diet, stress, hormonal influences, or abnormal menstrual cycles. Adult women with thinning hair were randomized to receive MPS (*N* = 30) or placebo (*N* = 30) twice daily for 90 days. Digital images were obtained from a 4 cm^2^ area scalp target area. Each subject's hair was washed and shed hairs were collected and counted. After 90 days, these measures were repeated and subjects completed Quality of Life and Self-Assessment Questionnaires. MPS-treated subjects achieved a significant increase in the number of terminal hairs within the target area (*P* < 0.0001) which was significantly greater than placebo (*P* < 0.0001). MPS use also resulted in significantly less hair shedding (*P* = 0.002) and higher total Self-Assessment (*P* = 0.006) and Quality of Life Questionnaires scores (*P* = 0.035). There were no reported adverse events. MPS promotes hair growth and decreases hair loss in women suffering from temporary thinning hair. This trial is registered with ClinicalTrials.gov Identifier: NCT02297360.

## 1. Introduction

Hair loss in women is often an overlooked and underappreciated condition that affects women almost as frequently as it affects men. In the United States, 40% of people with hair loss are women and 40% of women have visible hair loss by age of 40. Hair loss may begin in women as early as their teens [1] or 20s [2] and increases significantly with age. In one study, 6% of women aged under 50 years were diagnosed as having female pattern hair loss, increasing to 38% in subjects aged 70 years and over [3].

Reasons for female pattern hair loss include medical conditions, medications, and physiologic or emotional stress [4–6]. A recent study of twins showed that significant factors associated with hair loss in women included divorce or separation, multiple marriages, more children, longer sleep duration, higher stress severity, smoking, higher income, and various medical conditions [7]. Regardless of the cause, the psychological impact of hair loss in women is well-known [8–11]. The psychological impact of hair loss is also more severe for women than men.

Nutritional deficiencies are also a known cause of hair loss [4] and may include inadequate intake of proteins, minerals, essential fatty acids, and vitamins [12]. Dietary zinc deficiency may be a cause of alopecia [13, 14]. Compared to normal controls, serum zinc concentrations were shown to be significantly lower among women with female pattern hair loss [15], which can be halted or improved with zinc supplements [14, 16]. Zinc supplements can increase hair growth in patients with alopecia areata [17]. Hair loss in women has also been reported to be associated with iron deficiency [12–14]; however, the results of a large controlled study indicate that the incidence of iron deficiency among women with female pattern hair loss is not different than women with no hair loss [15]. Deficiencies in other minerals such as selenium may also be a factor in hair loss [18]. Biotin is a water-soluble vitamin and essential cofactor for several important enzymes [18]. Dietary deficiency of this important cofactor has also been associated with hair loss [13, 18, 19].

Currently, many medical treatments are available to men that are too dangerous for women or are unsafe for use in women of child-bearing age, such as finasteride [20]. Viviscal Extra Strength is an oral marine protein supplement (MPS) specifically designed to promote hair growth in women suffering from temporary thinning hair. MPS is a proprietary blend of shark and mollusc powder derived from sustainable marine sources which provides essential nutrients to nourish hair naturally and improve the appearance of thinning hair. It does not contain hormones or other drugs. MPS or the same ingredients contained in MPS were initially shown to have beneficial effects on hereditary androgenic alopecia in men [21] and photodamaged skin in women [22, 23]. The product has been marketed in Europe for over 15 years.

The purpose of this double-blind, placebo-controlled study was to further evaluate the ability of MPS to strengthen and promote the growth of terminal hairs in adult women with self-perceived thinning hair associated with poor diet, stress, hormonal influences, or abnormal menstrual cycles. We use the term hair loss to nonspecifically describe our study population as women who may notice their hair thinning even if it is not significantly dramatic enough for clinical diagnosis of true alopecia. True alopecia cases including alopecia areata, scarring alopecia, androgenetic alopecia, and telogen effluvium as described in our exclusion criteria were not included. Since female pattern hair loss is also characterized by changes in hair diameter associated with hair follicle miniaturization [24, 25], the effect of MPS on hair diameter will also be measured. Finally, this study also assessed the ability of MPS to decrease hair shedding.

## 2. Methods

### 2.1. Study Subjects

Women 21–65 years of age with Fitzpatrick photo skin types I–IV and self-perceived thinning hair associated with poor diet, stress, hormone influences, or abnormal menstrual cycle were eligible for enrollment. Each subject expressed her willingness to follow study procedures including maintaining their normal hair shampooing and color treatment frequency and not substantially changing their current diet, medications, or exercise routines for the duration of the study. Women of child-bearing potential were required to use a medically sound, nonhormonal form of birth control during the study.

Reasons for exclusion from study participation included a history of intolerance or allergy to fish, seafood, shellfish, or acerola; known allergy or sensitivity to any shampoo or conditioner; a stressful incident within the last 6 months, such as the death of a family member; use of hormones for birth control or hormone replacement therapy during the past 6 months; use of other therapies for hair growth such as light therapy or minoxidil within the last 3 months; use of medications known to affect the hair growth cycle within the last 6 months such as hormone-based contraceptives, cyproterone acetate, aldactone/spironolactone, finasteride, or other 5-alpha-reductase inhibitors; self-reported active hepatitis, immune deficiency, HIV, or autoimmune disease; self-reported uncontrolled diseases such as diabetes or hypertension; an active dermatologic condition which might place the subject at risk or interfere with the objectives of the study; other hair loss disorders, such as alopecia areata, scarring alopecia, androgenetic alopecia, and telogen effluvium; participating in any clinical research study; or nursing, pregnancy, or planning to become pregnant during the study.

### 2.2. Test Material

Subjects were randomized in double-blind fashion to receive the oral MPS (Viviscal Extra Strength Oral Tablets; Lifes2good, Inc., Chicago, IL) or placebo. The key ingredient in MPS tablets is AminoMar marine complex,* Equisetum arvense *sp. (horsetail which contains a naturally occurring form of silica),* Malpighia glabra *(acerola cherry which provides vitamin C), biotin, and zinc. The AminoMar is composed of a proprietary blend of shark powder and mollusc powder derived from sustainable marine sources. The placebo treatment consisted of inert tablets with similar appearance.

Subjects were instructed to take one tablet of their assigned treatment twice daily in the morning and evening following a meal. They were to maintain their normal hair care routine and use the same brand/type of hair care products and maintain the same haircut, color, and style for the study duration. Subjects with color-treated hair were also instructed to have the color treatment performed at the same time interval prior to each visit (if the color treatment was done 1 week prior to Visit 1, it should be repeated 1 week prior to Visit 2). Subjects were instructed to wash their hair at home 24 hours in advance of each study visit.

### 2.3. Study Procedures

This study consisted of a baseline clinic visit (Visit 1) and a follow-up visit at 90 days (Visit 2). A basic physical examination was performed at both visits which included a basic body systems overview, vital signs, and scalp examination to rule out any confounding scalp conditions. Subjects were instructed to wash their hair at home 24 hours in advance of each study visit. During the clinic visit, each subject had their hair washed with a commercial shampoo (Viviscal Gentle Shampoo; Lifes2good, Inc., Chicago, IL) over a sink containing a cheesecloth positioned to collect any shed hairs which were collected and counted.

During the baseline visit, an approximately 2 cm × 2 cm (4 cm^2^) area of the scalp was selected along the frontalis bone where frontal hairline and lateral hairline meet (hairline junction). A three-point location was recorded based on measurements obtained from the medial canthus, lateral canthus, and preauricular skin pit to the hairline junction. Where these three points meet (anterior lateral triangle of scalp), a target area 1 cm posterior into scalp hair was chosen. The 4 cm^2^ target area was marked using a black fine-tip skin marker such that the three-point triangulation mark was in the center.

Phototrichograms were obtained of the scalp target area at both visits using two-dimensional digital and macrophotographs (Canon SD 4500 IS and Nikon Coolpix 4300 cameras, resp., with a 3GEN Dermlite Foto37 system). All photographs were taken by the same staff member, in the same location, and with the same lighting. Ten [10] terminal hairs in the target area were randomly chosen throughout the area and cut at the surface of the scalp. Digital photographs were obtained to measure hair diameter 1 mm from the cut end of the hair (Dino-Lite Microscope; AnMo Electronics Corp., Torrance, CA). The hair diameter was measured and used to obtain an average hair diameter for the target area.

### 2.4. Study Endpoints

Using phototrichograms, the primary efficacy endpoint was the change in the number of terminal hairs and vellus hairs in the target area of the scalp at Visit 2. Terminal hairs were defined as short or long coarse hairs found on the scalp with a cross-sectional diameter of 40 *μ*M or greater. Vellus hairs were defined as fine, short hairs with a maximum cross-sectional diameter of less than 40 *μ*M. The secondary endpoints were changes in hair diameter, the number of shed hairs, and the responses to Quality of Life and Self-Assessment Questionnaires at Visit 2. Safety endpoints were changes in physical examinations, scalp condition, and vital signs. Subjects were queried about potential adverse events at Visit 2.

### 2.5. Statistical Analysis

Descriptive statistics were obtained for all variables. Tests of normality of continuous measures were made and data were examined for homogeneity of variance. Changes in baseline hair growth, hair diameter, hair shedding counts with hair washing, Quality of Life responses, and Self-Assessment Questionnaire responses were tested using analyses of variance with repeated measurements. All statistical tests were two-tailed. Differences were considered statistically significant at the level of *P* value of ≤0.05 obtained.

### 2.6. Ethics

This study protocol and informed consent forms were approved by an institutional review board (IRB Company Inc., Buena Park, CA). Each subject provided informed consent prior to participating in any study-related activity. This study strictly adhered to all applicable guidelines for the protection of human subjects for research as outlined in the United States FDA 21 CFR Part 50, in accordance with the accepted standards for good clinical practices and the standard practices of Ablon Skin Institute Research Center.

## 3. Results

### 3.1. Subject Enrollment

The study enrolled 60 women with a mean (SD) age of 48.6 years (10.0 years) (range, 24–65 years). Subjects were randomized to receive treatment with MPS (*N* = 30) or placebo (*N* = 30) and all subjects completed the study. The race/ethnicity of subjects was Caucasian (*N* = 53; 88%), Hispanic (*N* = 6; 10%), and Asian (*N* = 1; 2%). There were no significant differences between the two groups with respect to age (MPS: 50.2 ± 12 years; placebo: 46.9 ± 9 years; *f*_1,58_ = 1.58; *P* = 0.214) or race/ethnicity (MPS: Caucasian (*N* = 25; 83.3%); placebo: Caucasian (*N* = 28; 93.3%), df = 2, *P* = 0.399).

### 3.2. Primary Endpoints

**Figure 1:**
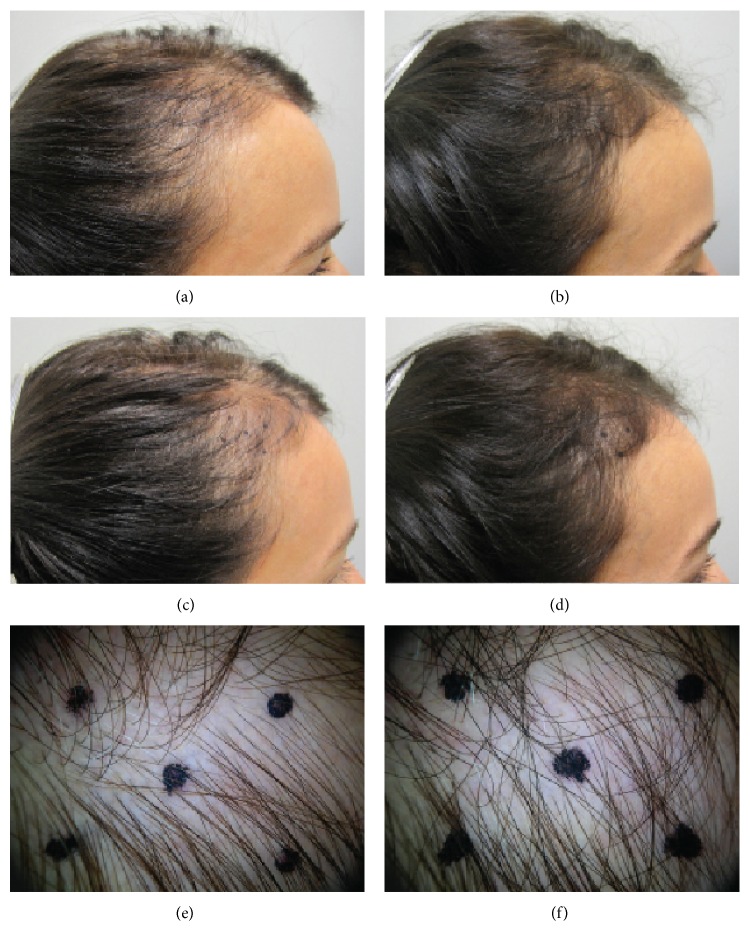
Subject 42. The number of terminal and vellus hairs at baseline (left) increased significantly after 90 days of treatment (right). The 4 cm^2^ target area of the scalp is shown in the middle and bottom rows.

**Figure 2:**
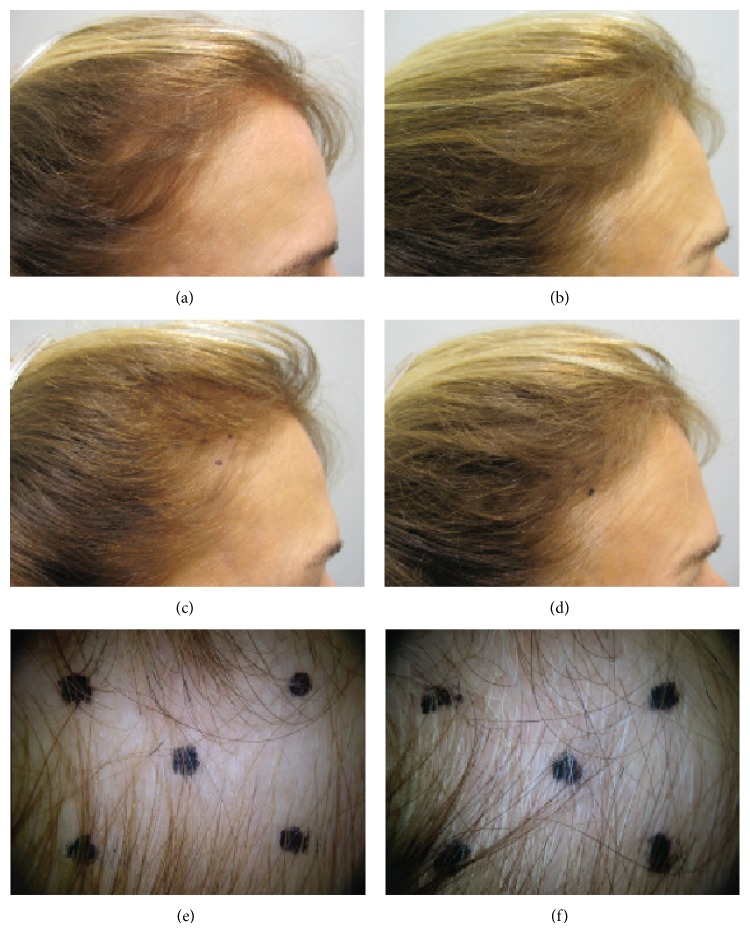
Subject 47. The number of terminal and vellus hairs at baseline (left) increased significantly after 90 days of treatment (right). The 4 cm^2^ target area of the scalp is shown in the middle and bottom rows.

Among the MPS-treated subjects, there was a significant increase in the mean number of terminal hairs from 178.3 (7.8) at baseline to 235.8 (18.4) at Visit 2 (*f*_(1,29)_ = 362.0, *P* < 0.0001) but not among placebotreated subjects from 178.2 (9.6) to 180.9 (18.8) (*f*_(1,29)_ = 1.25, *P* = 0.273) (Table 1). Consequently, the number of terminal hairs among MPS-treated subjects was also significantly greater than placebo-treated subjects at Visit 2 (*f*_(1,58)_ = 200.4, *P* < 0.0001) due to the greater increase among the MPS-treated subjects (MPS: D0:178.3 ± 8; D90:235.8 ± 18; placebo: D0:178.2 ± 10, D90:180.9 ± 18). Changes in the clinical appearance of two treated subjects at baseline and after 90 days of treatment are apparent in Figures 1 and 2.

Similarly, there was a significant increase in the number of vellus hairs in MPS-treated subjects (*f*_(1,29)_ = 54.1, *P* < 0.0001) but not among the placebotreated subjects (*f*_(1,29)_ = 0.67, *P* = 0.420) (Table 1). The mean number of vellus hairs in the MPS group at Visit 2 was again significantly greater than the placebo group. The two groups were significantly different in the number of vellus hairs (*f*_(1,58)_ = 24.21, *P* < 0.0001) due to the significant increase among the MPS-treated subjects (MPS: D0:19.57 ± 2; D90:21.23 ± 2; placebo: D0:19.8 ± 2, D90:19.97 ± 2).

### 3.3. Secondary Endpoints

The two groups were significantly different in the hair shedding counts following hair washing (*f*_(1,58)_ = 4.51, *P* = 0.038) due to significant decrease among the MPS-treated subjects (MPS: D0: 27.13 ± 27; D90:16.47 ± 14; placebo: D0:23.4 ± 25, D90:21.87 ± 21). There was no significant increase in terminal hair diameter among MPS-treated subjects (*f*_(1,29)_ = 11.42, *P* = 0.434) or placebotreated subjects (*f*_(1,29)_ = 0.29, *P* = 0.725) and the two groups were not significantly different from one another with respect to terminal hair diameter (*f*_(1,58)_ = 0.674, *P* = 0.415).

Subjects treated with MPS obtained significantly higher total scores on the Self-Assessment Questionnaire at Visit 2 (*f*_(1,58)_ = 8.27, *P* = 0.006; MPS: 61.20 ± 7; placebo: 55.57 ± 8) with significant differences between the two groups on 7 of 13 items including overall hair growth, overall hair volume, scalp coverage, thickness of hair body, hair strength, growth of eyebrow hair, and overall skin health (Table 2).

The MPS-treated subjects also obtained significantly higher total scores on the Quality of Life Questionnaire (*f*_(1,58)_ = 4.61, *P* = 0.035; MPS: D0:33.9 ± 10; D90:26.9 ± 8; placebo: D0:34.0 ± 11, D90:30.6 + 11). These differences were significant within each group (MPS: *f*_(1,29)_ = 24.00, *P* < 0.001; placebo: *f*_(1,29)_ = 13.8, *P* < 0.001); however, scores for MPS-treated subjects were significantly higher on all 15 questionnaire items but only 10 for the placebo-treated subjects (Table 3).

## 4. Discussion

The results of this study demonstrate that the use of MPS tablets for 90 days increased the number of terminal hairs and decreased hair shedding in women with self-perceived thinning hair. These changes were associated with improved hair quality including overall hair growth and increased hair strength. There were no reports of adverse events.

Other US studies have demonstrated the beneficial effects of MPS when used by women with thinning hair. In a placebo-controlled, double-blind pilot study, adult women with self-perceived thinning hair were randomized to receive MPS (*N* = 10) or placebo (*N* = 5) twice daily [26]. After 180 days, the mean (SD) number of terminal vellus hairs among placebotreated subjects at baseline was 256.0 (24.1), remaining at 245.0 (22.4) and 242.2 (26.9) after 90 and 180 days, respectively. In contrast, the mean number of baseline terminal hairs in MPS-treated subjects was 271.0 (24.2) increasing to 571 (65.7) and 609.6 (66.6) after 90 and 180 days, respectively (for each, *P* < 0.001* versus* placebo). Subjects also reported improvements in several subjective measures of hair quality.

A randomized, double-blind, multicenter, placebo-controlled study was also designed to assess the effects of MPS on hair growth in adult women with self-perceived thinning hair (Ablon and Dayan, submitted). These subjects were randomized to receive MPS (*N* = 20) or placebo (*N* = 20) twice daily. The MPS-treated subjects achieved a significant increase in the number of baseline terminal hairs at 90 and 180 days (for each, *P* < 0.0001) and were significantly greater than placebo (*P* < 0.0001). These objective measures were correlated with numerous improvements in Self-Assessment and Quality of Life measures.

The results of this study are also in agreement with another study which showed the use of an oral supplement containing natural ingredients including marine-derived protein (shark cartilage) and fish oil (omega-3 polyunsaturated fatty acids) significantly reducing hair loss in women [27]; however, it did not promote hair growth.

## 5. Conclusion

Similar to previous studies, the ingredients in MPS tablets promote hair growth in women suffering from temporary thinning hair. The current study further demonstrated the ability of this product to decrease hair loss. MPS continues to demonstrate an excellent safety profile.

## Figures and Tables

**Table 1:** Changes in hair growth and hair diameter.

MPS, *N* = 30	Baseline	Day 90	Significance^*^

Mean terminal hair count (SD)	178.3 (7.8)	235.8 (18.4)	*P* < 0.0001
Mean vellus hair count (SD)	19.6 (2.1)	21.2 (2.2)	*P* < 0.0001
Mean terminal hair diameter, mm (SD)	0.062 (0.009)	0.063 (0.009)	*P* = NS
Mean shed hair counts (SD)	27.1 (26.6)	16.5 (14.4)	*P* = 0.002

Placebo, *N* = 30	Baseline	Day 90	Significance^*^

Mean terminal hairs (SD)	178.2 (9.6)	180.9 (18.8)	*P* = NS
Mean vellus hairs (SD)	19.8 (1.7)	20.0 (1.9)	*P* = NS
Mean terminal hair diameter, mm (SD)	0.063 (0.009)	0.062 (0.008)	*P* = NS
Mean shed hair counts (SD)	23.4 (25.5)	21.9 (20.8)	*P* = NS

^*^Repeated measures ANOVA across study days contrasts per treatment group; NS, not significant. Baseline versus 90 days.

**Table 2:** Self-Assessment Questionnaire results.

Quality	MPS, *N* = 30	Placebo, *N* = 30	Significance^*^
	Day 90	Day 90	
(1) Overall hair growth	5.03 (0.85)	4.53 (0.68)	*P* = 0.015
(2) Overall hair volume	4.97 (0.72)	4.17 (0.79)	*P* < 0.0001
(3) Scalp coverage	4.70 (0.14)	4.23 (0.97)	*P* = 0.042
(4) Thickness of hair body	4.77 (0.82)	4.20 (0.89)	*P* = 0.013
(5) Softness of hair body	4.67 (0.96)	4.33 (0.84)	*P* = NS
(6) Hair shine	4.60 (1.00)	4.37 (0.77)	*P* = NS
(7) Hair strength	4.97 (1.00)	4.33 (0.92)	*P* = 0.013
(8) Nail strength	4.97 (1.03)	4.47 (1.11)	*P* = NS
(9) Nail growth rate	4.73 (0.98)	4.40 (0.89)	*P* = NS
(10) Growth of eyebrow hair	4.40 (0.86)	4.00 (0.53)	*P* = 0.033
(11) Growth of eyelashes	4.27 (0.69)	4.17 (0.59)	*P* = NS
(12) Skin smoothness	4.50 (0.82)	4.20 (0.81)	*P* = NS
(13) Overall skin health	4.63 (0.85)	4.17 (0.91)	*P* = 0.045

^*^One-way analysis of variance; NS, not significant.

**Table 3:** Quality of Life Questionnaire results.

Question	MPS, *N* = 30			Placebo, *N* = 30		
	Baseline	Day 90	Significance^*^	Baseline	Day 90	Significance^*^
(1) I am embarrassed by my thinning hair.	2.70 ( 0.84)	2.13 (0.82)	*P* < 0.0001	2.73 (1.02)	2.47 (0.92)	*P* = 0.009
(2) Because of my thinning hair, I avoid social gatherings.	1.77 (0.68)	1.37 (0.49)	*P* = 0.001	1.67 (0.84)	1.53 (0.78)	*P* = NS
(3) I do not like meeting new people as I feel they are judging me because of my thinning hair.	2.10 (0.76)	1.63 (0.62)	*P* = 0.001	1.87 (0.94)	1.73 (0.87)	*P* = NS
(4) I avoid going out during the day because of my thinning hair.	1.77 (0.68)	1.37 (0.49)	*P* = 0.001	1.70 (0.92)	1.50 (0.78)	*P* = 0.012
(5) My condition impacts my emotional state at work.	2.10 (0.92)	1.73 (0.74)	*P* = 0.005	2.13 (0.90)	2.03 (1.03)	*P* = NS
(6) I feel my thinning hair has impacted my ability to succeed in interviews.	2.07 (0.74)	1.57 (0.94)	*P* = 0.007	1.73 (1.14)	1.60 (1.04)	*P* = NS
(7) My condition has prevented my participation in a sports activity.	2.07 (0.94)	1.57 (0.77)	*P* < 0.0001	1.83 (0.95)	1.83 (0.75)	*P* = NS
(8) My condition impacts my self-esteem.	2.87 (0.86)	2.20 (0.81)	*P* < 0.0001	2.97 (0.93)	2.53 (0.97)	*P* = 0.002
(9) My condition makes me feel self-conscious about my thinning hair.	3.03 (0.81)	2.43 (0.77)	*P* < 0.0001	3.13 (0.90)	2.80 (1.03)	*P* = 0.01
(10) Because of my thinning hair, I fear being the center of attention.	2.00 (0.91)	1.57 (0.77)	*P* = 0.01	2.37 (1.00)	2.10 (1.24)	*P* = 0.043
(11) I feel my thinning hair is affecting my personal relationships.	2.23 (0.97)	1.87 (0.86)	*P* = 0.014	2.33 (1.09)	2.03 (1.03)	*P* = 0.037
(12) Because of my thinning hair, I avoid being intimate with my partner.	1.70 (0.92)	1.37 (0.67)	*P* = 0.039	1.67 (0.92)	1.47 (0.86)	*P* = NS
(13) Because of my thinning hair, I feel less attractive to my significant other.	2.47 (1.01)	1.93 (1.05)	*P* = 0.001	2.73 (1.11)	2.47 (1.20)	*P* = 0.009
(14) Because of my thinning hair, I am less outgoing than I would like to be.	2.33 (1.03)	1.90 (0.92)	*P* = 0.001	2.27 (0.94)	2.02 (1.07)	*P* = 0.032
(15) Because of my thinning hair, I feel unattractive.	2.70 (1.02)	2.27 (0.94)	*P* = 0.001	2.87 (0.90)	2.50 (0.97)	*P* = 0.001

^*^Repeated measures ANOVA across study days contrasts per treatment group; NS, not significant.
